# Custos do Tratamento Clínico de Pacientes com Insuficiência Cardíaca Avançada na América Latina

**DOI:** 10.36660/abc.20240813

**Published:** 2025-11-19

**Authors:** Livia Adams Goldraich, Ana Paula Beck da Silva Etges, Laura Caroline Tavares Hastenteufel, Dayanna Machado Lemos, Andreas Zuckermann, Mandeep R. Mehra, Carisi Anne Polanczyk, Nadine Clausell

**Affiliations:** 1 Programa de Insuficiência Cardíaca Avançada e Transplante Cardíaco Hospital de Clínicas de Porto Alegre Porto Alegre RS Brasil Programa de Insuficiência Cardíaca Avançada e Transplante Cardíaco, Hospital de Clínicas de Porto Alegre, Porto Alegre, RS – Brasil; 2 Programa de Pós-Graduação em Cardiologia Faculdade de Medicina Universidade Federal do Rio Grande do Sul Porto Alegre RS Brasil Programa de Pós-Graduação em Cardiologia, Faculdade de Medicina, Universidade Federal do Rio Grande do Sul, Porto Alegre, RS – Brasil; 3 Instituto Nacional de Ciência e Tecnologia para Avaliação de Tecnologias em Saúde Conselho Nacional de Desenvolvimento Científico e Tecnológico Porto Alegre RS Brasil Instituto Nacional de Ciência e Tecnologia para Avaliação de Tecnologias em Saúde e Conselho Nacional de Desenvolvimento Científico e Tecnológico, Porto Alegre, RS – Brasil; 4 Programa de Pós-Graduação em Epidemiologia Faculdade de Medicina Universidade Federal do Rio Grande do Sul Porto Alegre RS Brasil Programa de Pós-Graduação em Epidemiologia, Faculdade de Medicina, Universidade Federal do Rio Grande do Sul, Porto Alegre, RS – Brasil; 5 Department of Cardiac Surgery Medical University of Vienna Vienna Áustria Department of Cardiac Surgery, Medical University of Vienna, Vienna – Áustria; 6 Brigham and Women’s Hospital Harvard Medical School Boston MA EUA Brigham and Women’s Hospital and Harvard Medical School, Boston, MA – EUA

**Keywords:** Custos e Análise de Custo, Insuficiência Cardíaca, América Latina

## Abstract

**Fundamento:**

A carga financeira enfrentada por pacientes com insuficiência cardíaca (IC) avançada, que não são elegíveis para transplante cardíaco ou não têm acesso à terapia avançada com dispositivos de assistência ventricular esquerda (DAVEs) ainda não foi descrito na América Latina.

**Objetivo:**

Realizar um estudo de microcustos em pacientes que, embora elegíveis para terapia com DAVE, não tiveram acesso ao procedimento.

**Métodos:**

Avaliamos os custos diretos do cuidado em uma coorte de pacientes com IC avançada tratados em um hospital público brasileiro, que eram candidatos à terapia com DAVE (e inelegíveis para transplante), mas não tiveram acesso ao dispositivo. Foi realizada uma análise de custos em nível individual desde o momento em que a necessidade de DAVE foi identificada até o óbito ou o fim do acompanhamento. O custo total por paciente foi calculado utilizando a metodologia de custeio baseado em atividades com base no tempo ( *time-driven activity-based costing* ) e expresso em dólares internacionais (Int$). Os pacientes foram estratificados por gravidade da doença e mortalidade por todas as causas para avaliar a variabilidade dos custos.

**Resultados:**

Vinte pacientes consecutivos (idade média de 51 anos; 90% do sexo masculino) foram acompanhados por uma média de 15 meses, com uma taxa de sobrevida global de 40%. O custo médio por paciente foi de Int$120.457 (desvio padrão (DP) Int$78.029). As internações representaram a maior parcela dos custos totais, correspondendo a 72% (Figura 2). Pacientes com maior gravidade clínica (perfil INTERMACS ≤ 3) apresentaram custos significativamente mais elevados (Int$156.457), quase 70% superiores aos daqueles que não necessitaram de suporte inotrópico (Int$91.003).

**Conclusões:**

Este estudo evidencia o impacto financeiro associado ao manejo clínico contínuo da IC avançada em pacientes que, de outra forma, seriam elegíveis para terapia com DAVE. Esses achados fornecem subsídios para avaliar a relação custo-efetividade de intervenções com dispositivos de suporte à vida em populações selecionadas com IC avançada.

## Graphical abstract

**Figura Central:**
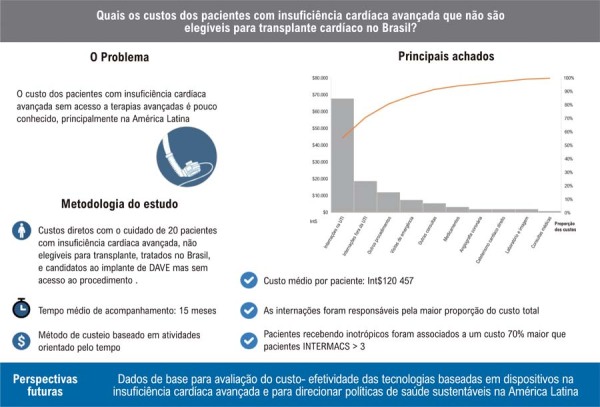
: Custos do Tratamento Clínico de Pacientes com Insuficiência Cardíaca Avançada na América Latina

## Introdução

A insuficiência cardíaca (IC) avançada, uma fase da doença caracterizada pela resistência às terapias farmacológicas e não farmacológicas tradicionais, está associada à alta morbidade e mortalidade, além de maior utilização de recursos de saúde e custos elevados tanto para os indivíduos quanto para a sociedade.^1^ À medida que as populações envelhecem e se beneficiam de terapias aprimoradas para doenças cardiovasculares de início precoce, a prevalência de IC avançada continua a aumentar, resultando em um impacto social significativo e perda de produtividade.^2^

Embora o transplante cardíaco seja atualmente uma terapia estabelecida para a IC avançada, um número substancial de indivíduos é inelegível devido a comorbidades ou outros fatores.^3^ Para esses pacientes, um dispositivo de assistência ventricular esquerda (DAVE) durável funciona como uma terapia que prolonga a vida; caso contrário, eles são frequentemente relegados aos cuidados paliativos.^4^ Nos Estados Unidos, 27298 DAVEs de longo prazo foram implantados entre 2010 e 2019; somente em 2019, o número de implantes de DAVE quase igualou o número de transplantes cardíacos realizados no país, com a maioria dos casos classificados como terapia de destino para pacientes considerados inelegíveis ao transplante.^5 , 6^ Na Europa, aproximadamente 500 indivíduos por ano receberam um DAVE nos últimos 10 anos, e esse número continua a crescer devido à escassez de órgãos doadores.^7^

Os desfechos para pacientes com DAVE de longo prazo melhoraram substancialmente ao longo do tempo, em grande parte devido à melhor seleção de pacientes, ao aprimoramento do manejo clínico e à introdução da bomba HeartMate 3 (Abbott, EUA), totalmente levitada magneticamente. A sobrevida mediana agora ultrapassa cinco anos, especialmente entre pacientes inelegíveis para transplante.^8 - 10^ No entanto, os DAVEs não estão disponíveis na maioria dos países de baixa e média renda devido a preocupações com a relação custo-efetividade. No Brasil, o DAVE HeartMate 3 é acessível apenas por meio de seguros privados ou iniciativas de pesquisa filantrópicas.^1 1^ O custo do cuidado de pacientes com IC avançada que não têm acesso à terapia com DAVE permanece amplamente desconhecido, apesar de sua potencial relevância para a avaliação da custo-efetividade dessas intervenções.

A utilização de recursos de saúde e o custo do cuidado para pacientes com IC avançada tratados sem possibilidade de resgate por meio de DAVE ou transplante podem representar um ônus financeiro que não é prontamente reconhecido por indivíduos, sistemas de saúde, pagadores e governos. Em casos selecionados, o uso criterioso da terapia com DAVE como destino final pode se mostrar custo-efetivo.^11 , 12^ Portanto, o presente estudo, conduzido dentro do sistema público de saúde brasileiro, tem como objetivo avaliar os custos longitudinais do manejo médico da IC avançada em pacientes que poderiam ter sido elegíveis para a terapia com DAVE como destino, mas que não tiveram acesso ao procedimento.

## Métodos

Realizamos um estudo de microcustos (um método de estimativa financeira que inclui o uso de recursos e o custo unitário) para avaliar os custos diretos de uma coorte de pacientes com IC avançada, sob a perspectiva de um hospital universitário público, em um centro regional de referência para terapias avançadas de IC. A amostra foi composta por pacientes com IC avançada que não eram elegíveis para transplante cardíaco, mas elegíveis para receber um DAVE como indicação de terapia de destino.^13^

### População

Definimos uma coorte de pacientes adultos consecutivos com IC em estágio avançado que atendiam aos critérios de elegibilidade para implantação de DAVE como terapia de destino, mas que não puderam receber esse tratamento em nossa instituição entre janeiro de 2015 e maio de 2023. A IC avançada foi definida por meio de avaliação clínica, incorporando carga de sintomas, parâmetros prognósticos objetivos e intervenções terapêuticas (como terapia inotrópica), conforme descrito pelas sociedades europeias e norte-americanas de cardiologia e resumido por Truby e Rogers.^14^ Os critérios de exclusão incluíram pacientes que foram submetidos a transplante cardíaco ou implantação de DAVE, aqueles com cobertura por plano de saúde privado e aqueles acompanhados principalmente em outras instituições.

**Figura 1:**
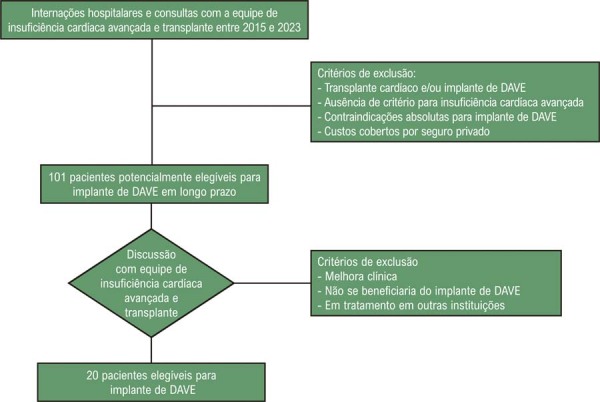
– Fluxograma de pacientes elegíveis para terapia com dispositivo de assistência ventricular esquerda (DAVE) de longo prazo no centro de referência selecionado para análise de custo.

As razões clínicas para inelegibilidade ao transplante foram idade avançada, hipertensão pulmonar grave, obesidade, tabagismo ativo, fragilidade e outras comorbidades. Nosso programa segue as diretrizes da *International Society for Heart and Lung Transplantation* quanto às contraindicações para o transplante.^15 , 16^ A hipertensão pulmonar grave foi definida como resistência vascular pulmonar superior a 3 unidades Wood, não responsiva ao teste com vasodilatadores. O fluxograma dos pacientes elegíveis para terapia com DAVE de longo prazo e selecionados para inclusão na análise de custos está na Figura 1 .

O período de coleta de dados para cada paciente começou quando a equipe clínica considerou a indicação de terapia avançada (cardíaco ou DAVE) ou na admissão hospitalar para aqueles que já estavam internados nessa data. O intervalo de aquisição de custos foi registrado no óbito, no final do acompanhamento do estudo em 30 de junho de 2023 ou na alta hospitalar para os pacientes internados nessa data. As fontes de dados incluíram resumos eletrônicos de alta, notas de evolução, registros de consultas ambulatoriais e formulários de avaliação para transplante cardíaco/DAVE. A análise de custos em nível individual incluiu todos os atendimentos hospitalares, incluindo consultas, exames diagnósticos, procedimentos e internações.

### Dados de custo

Os componentes de custo para a análise geral foram obtidos utilizando a metodologia de custeio baseado em atividades com base no tempo.^17^ Nessa abordagem, o consumo de recursos é medido com base no ciclo de cuidados ao paciente, considerando tanto o custo unitário da prestação do serviço quanto o tempo necessário para realizar cada transação ou atividade. Os dados de custo relacionados a pessoal, infraestrutura, procedimentos e exames diagnósticos (incluindo imagem) foram extraídos dos registros administrativos do hospital, seguindo a metodologia utilizada em estudos anteriores focados em procedimentos específicos e serviços prestados a pacientes com IC avançada ( Tabela 1 ).

**Tabela 1:** – Fontes de informação dos componentes de custo com valores atualizados para o ano de 2023

Profissional da saúde	Salários mais encargos (Int$/h)	Fonte de informação
Cardiologista clínico	73	Departamento financeiro da instituição
Cardiologista intervencionista	86	Departamento financeiro da instituição
Residente médico	6	Departamento financeiro da instituição
Cardiologista especializado em imagem	82	Departamento financeiro da instituição
Cirurgião cardiovascular	82	Departamento financeiro da instituição
Enfermeira	38	Departamento financeiro da instituição
Técnico de enfermagem	21	Departamento financeiro da instituição
Fisioterapeuta	36	Departamento financeiro da instituição
Perfusionista	31	Departamento financeiro da instituição
Anestesiologista	93	Departamento financeiro da instituição
**Infraestrutura nas unidades de internação**		
Unidade	Custos fixos atribuídos à unidade (Int$)	Fonte de informação
Laboratório de cateterismo	433 (hora)	Departamento financeiro da instituição
Unidade de cuidados coronarianos	2178 (dia)	Departamento financeiro da instituição
Unidade de terapia intensiva	2969 (dia)	Departamento financeiro da instituição
Enfermaria de internação	349 (dia)	Departamento financeiro da instituição
Procedimentos e serviços *		
Procedimento e/ou serviço	Custo (Int$ por unidade)	Fonte da informação
Inserção de CCIP	638	[28]
Inserção de cateter venoso central	259	[28]
Consulta ambulatorial para paciente classe II da NYHA **	176	[29]
Consulta ambulatorial para paciente classes III e IV da NYHA **	593	[29]
Visita ao hospital-dia	76	[30]
Sessão de hemodiálise	488	Departamento financeiro da instituição
Angioplastia coronariana	Materiais 1664 + Professionais e espaço físico 1407	Departamento financeiro da instituição
Cateterismo cardíaco esquerdo	Materiais 240 + Professionais e espaço físico 390	Departamento financeiro da instituição
Cateterismo cardíaco direito	Materiais 484 + Professionais e espaço físico 445	Departamento financeiro da instituição
Implantação de balão intra-aórtico	Materiais 1281 + Professionais e espaço físico 678	Departamento financeiro da instituição
Biópsia endomiocárdica	Materiais 743+ Professionais e espaço físico 390	Departamento financeiro da instituição
Implante de desfibrilador cardioversor implantável	Materiais + 11983 Professionais e espaço físico 1609	Departamento financeiro da instituição
Cardioversão elétrica	Materiais 33 + Professionais e espaço físico 234	Departamento financeiro da instituição
Reparo mitral transcateter borda a borda (TEER)	Materiais 62015 + Professionais e espaço físico 2363	Departamento financeiro da instituição

Int$: dólar internacional; CCIP: Cateter Central de Inserção Periférica; NYHA: New York Heart Association; * Tabela Suplementar I descreve o tempo dedicado pelos profissionais e custo com procedimentos individuais. ** Consultas ambulatórias incluem o gasto com exames realizados no mesmo dia.

Os custos com medicamentos foram obtidos a partir de dados de consumo em nível individual de pacientes, extraídos do sistema administrativo institucional. Os custos dos procedimentos incluíram materiais, honorários profissionais e infraestrutura. Foram realizadas entrevistas para estimar o tempo médio que os profissionais de saúde dedicaram a cada procedimento, consulta, atendimento ambulatorial e cuidados em hospital-dia. Os custos das unidades hospitalares — como enfermarias, Unidades de Terapia Intensiva (UTIs) e laboratório de cateterismo — englobaram depreciação, energia, materiais administrativos, salários profissionais e outras despesas. Para os salários profissionais, foi calculada a alocação exata de pessoal para cada unidade, incluindo a área da UTI especificamente destinada a pacientes cardiológicos, que possui uma estrutura de pessoal diferenciada. O custo fixo direto médio mensal de cada unidade foi calculado e dividido pela capacidade mensal de atendimento da respectiva unidade. Por exemplo, para a UTI, a capacidade foi determinada com base no número de leitos disponíveis: 45 leitos operando 24 horas por dia, durante 30 dias, totalizando 32.400 horas. O custo diário médio foi calculado dividindo-se o custo total atribuído à unidade pelo número de leitos e dias úteis. Os custos de internação foram então determinados multiplicando o tempo que cada paciente permaneceu em determinada unidade pelo respectivo custo por unidade de tempo.

Os componentes de custo foram calculados e agregados para determinar o custo total por paciente. As análises descritivas de custo incluíram média, DP, mediana e intervalo interquartil. Os pacientes foram estratificados por variáveis clínicas – especificamente, pela gravidade da doença conforme os perfis INTERMACS (Registro Interagências para Suporte Circulatório Mecânico) — e pelo desfecho de mortalidade, com o objetivo de estimar a variabilidade dos custos. Além disso, foi calculado o custo anual estimado por paciente para representar o impacto econômico esperado anual dessa condição clínica para os prestadores de serviços de saúde. Como a coorte do estudo incluiu pacientes com diferentes períodos de acompanhamento, o custo total por paciente foi dividido pelo número de meses de seguimento e, em seguida, multiplicado por 12. É importante considerar esse ajuste ao aplicar os dados em modelos econômicos futuros. Todos os valores foram atualizados para o ano de 2023 utilizando o Índice Nacional de Preços ao Consumidor Amplo (IPCA), acumulado desde a data original de obtenção dos dados (para os custos extraídos de artigos científicos). Esses valores são apresentados em dólares internacionais (Int$) para refletir a paridade do poder de compra, com base no fator de conversão do Banco Mundial disponível em https://data.worldbank.org/indicator/PA.NUS.PPP (acesso em 29 de março de 2024). Como referência, Int$ 1.000 equivale a US$ 1.000 e R$ 2.580. As comparações entre os perfis INTERMACS > 3 e INTERMACS ≤ 3, bem como entre os desfechos (vivo vs. óbito), foram realizadas utilizando o teste t de Student. Todas as análises foram realizadas utilizando o Microsoft^®^ Excel para Mac.

### Aspectos éticos

O protocolo de pesquisa foi aprovado pelo Comitê Institucional de Pesquisa e Ética, e o consentimento informado foi dispensado devido à natureza retrospectiva do estudo. Todos os pesquisadores assinaram um termo de confidencialidade para acessar os dados individuais. O estudo foi conduzido de forma independente e com total autonomia pelos autores, sem qualquer interferência do patrocinador.

## Resultados

Os critérios de inclusão e exclusão foram atendidos em 20 pacientes consecutivos com IC avançada. A idade média foi de 51 anos, e 90% eram do sexo masculino. A Tabela 2 apresenta as características demográficas e clínicas da coorte do estudo. No momento da inclusão, três quartos dos pacientes estavam hospitalizados, e quase metade apresentava status INTERMACS ≤ 3 — que representa graus de estabilidade sob terapia inotrópica, variando do menos (1) ao mais (3) estável — quando um DAVE como terapia de destino foi considerado, mas não pôde ser implantado. Na inclusão do estudo, a proporção de pacientes em terapia medicamentosa guiada por diretrizes para IC era relativamente baixa, principalmente devido à baixa tolerabilidade. Os inibidores de SGLT2 não estavam amplamente acessíveis no Brasil para tratamento da IC antes de 2022, o que resultou em baixas taxas de uso; além disso, sua utilidade na IC avançada permanece incerta, já que esses pacientes geralmente não foram incluídos nos principais estudos clínicos.^18^

**Tabela 2:** – Características demográficas e clínicas do da coorte do estudo

Características demográficas e comorbidades	N = 20
Sexo masculino	18 (90)
Idade, anos	51 ± 15
Diabetes	8 (40)
Fibrilação ou *flutter* atrial	6 (30)
Creatinina, mg/dL	1,5 ± 0,6
**Características da insuficiência cardíaca**	
Etiologia isquêmica	13 (65)
Fração de ejeção ventricular esquerda	0,21 [0,20- 0,24]
INTERMACS ≤ 3	9 (45)
Índice cardíaco, L/min/m^2 a^	2,2 ± 0,4
Resistência vascular pulmonar, unidade Wood ^b^	3,6 ± 2,1
PSAP, mmHg	56 ± 19
**Medicamentos**	
Inibidor de ECA / BRA	4 (20)
Inibidor de neprilisina	7 (35)
Betabloqueador	15 (75)
Antagonista mineralocorticoide	14 (70)
Inibidor de SGLT2	5 (25)
Inotrópico intravenoso	9 (45)
Tempo mediano de acompanhamento, em meses	15 [7,7 - 21,7]

Dados expressos em número (porcentagem), média ± desvio padrão ou mediana [intervalo interquartil]. INTERMACS: Interagency Registry for Mechanically Assisted Circulatory Support; BRA: bloqueadores dos receptores da angiotensina; PSAP: pressão sistólica da artéria pulmonar; ECA: enzima conversora de angiotensina; SGLT2: cotransportador sódio-glicose 2. ^a^ Dados disponíveis de 16 pacientes; ^b^ Dados disponíveis de 17 pacientes.

A principal contraindicação para a inelegibilidade ao transplante cardíaco foi a hipertensão pulmonar, seguida pelas comorbidades. A sobrevida global foi de 40% ao final de uma mediana de 15 meses (mínimo de quatro, máximo de 36) de seguimento; onze pacientes evoluíram para óbito devido à falência progressiva da bomba, e um por morte súbita. As características clínicas de gravidade dos pacientes individuais e as informações sobre utilização de recursos estão descritas na Tabela 3 .

**Tabela 3:** – Características clínicas de gravidade e dados de utilização de recursos por paciente

Paciente	Sexo	Idade, anos	Status INTERMACS	Condições de risco para transplante	Ano da indicação do DAVE	Tempo de seguimento, meses	Status ao final do seguimento	Visitas de emergência e/ou internações	Admissão na UTI, dias	Admissão na enfermaria e/ou na emergência, dias
P1	M	24	5	Hipertensão pulmonar	2020	31	Óbito	0	36	109
P2	M	66	4	Obesidade	2020	36	Vivo	0	12	20
P3	M	69	5	Hipertensão pulmonar	2022	15	Vivo	2	32	123
P4	M	61	3	Hipertensão pulmonar	2021	5	Óbito	0	8	139
P5	M	37	5	Hipertensão pulmonar	2022	8	Vivo	0	7	11
P6	M	66	5	Esternotomia prévia, PAOD	2022	8	Vivo	0	1	12
P7	M	43	3	Hipertensão pulmonar	2023	7	Vivo	0	88	120
P8	M	50	3	Hipertensão pulmonar	2020	4	Óbito	0	24	31
P9	M	39	3	Hipertensão pulmonar	2015	9	Óbito	0	44	56
P10	M	55	2	Hipertensão pulmonar	2022	20	Vivo	0	45	126
P11	M	64	4	DPCO, DAOP	2020	14	Óbito	2	1	28
P12	M	51	2	Hipertensão pulmonar	2018	6	Óbito	0	63	2
P13	F	49	3	Tabagista ativo	2017	11	Óbito	1	28	82
P14	M	48	3	Hipertensão pulmonar	2022	15	Vivo	0	23	12
P15	M	14	3	Hipertensão pulmonar	2020	4	Óbito	0	120	0
P16	F	63	5	Fragilidade, DM mal controlada	2019	34	Óbito	0	18	92
P17	M	31	6	Pneumonite relacionada à cannabis	2021	11	Vivo	0	0	0
P18	M	60	5	Obesidade, DM mal controlada	2017	27	Óbito	0	5	32
P19	M	58	4	Fragilidade, DPOC	2019	12	Óbito	0	36	76
P20	M	62	4	Hipertensão pulmonar	2016	32	Óbito	2	33	54

M: masculino; F: feminino; INTERMACS: Interagency Registry for Mechanically Assisted Circulatory Support; DAVE: dispositivo de assistência ventricular esquerda; UTI: unidade de terapia intensiva; DAOP: doença arterial obstrutiva periférica; DPOC: doença pulmonar obstrutiva crônica; DM: diabetes mellitus *P15 passou por uma única internação de longa duração desde o início da apresentação clínica.

### Análise de custo

**Figura 2:**
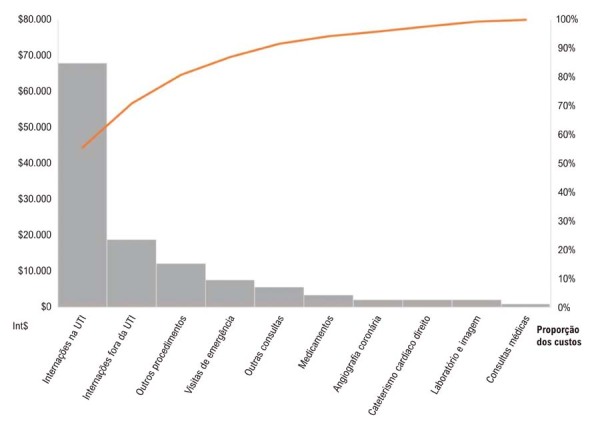
– Proporções dos componentes dos custos para a coorte de pacientes; UTI: unidade de terapia intensiva. Outros procedimentos incluem hemodiálise, transfusão de hemocomponentes, biópsias endomiocárdicas, implante de cardiodesfibrilador implantável e/ou terapia de ressincronização cardíaca, reparo mitral transcateter borda a borda, inserção de cateter venoso central periférico, angioplastia coronariana, cardioversão elétrica externa e implantação de balão intra-aórtico.

O custo médio por paciente foi de Int$ 120 457 (DP 78 029), o que representa um custo mensal médio de Int$ 8030. A Tabela 4 descreve a composição dos custos por paciente incluído no estudo, e a Figura 2 apresenta a proporção dessa composição. Os pacientes permaneceram, em média, 31 dias (DP 30) em UTIs e 53 dias (DP 47) em unidades não intensivas, entre a data da indicação do DAVE e o final do seguimento do estudo. As internações representaram mais de dois terços do custo total (56% em terapia intensiva e 16% em cuidados não intensivos). Medicamentos dispensados exclusivamente durante a internação corresponderam a 3% do custo médio total.

**Tabela 4:** – Descrição dos componentes de custo, custo total, e custo anual por paciente

Paciente	Medicamentos	Exames	Visitas à emergência	Admissão na enfermaria	Admissão na UTI	Consultas	Hospital-dia e ambulatórios	CCE	CCD	Outros procedimentos cardiovasculares e invasivos, hemodiálise e hemocomponentes	Custo total
P1	9146	2709	5937	37324	78407	1350	5507	0	2789	1277	144,446
P2	1401	2238	23750	4186	26136	696	13858	631	2789	13860	89,544
P3	5128	2653	26719	39765	69696	1545	6259	0	1859	15509	169,133
P4	0	0	0	48486	17424	1392	0	0	1859	1277	70,438
P5	326	849	2969	3488	15246	308	1727	0	2789	0	27,701
P6	87	756	0	4186	2178	180	3764	0	930	0	12,082
P7	4,413	1473	0	41858	191663	1622	2964	1263	2789	16338	264,381
P8	2,481	1809	0	10813	52272	738	0	0	1859	639	70,612
P9	4,683	3055	2969	19185	95831	1196	1778	631	1859	0	131,188
P10	9,119	4883	0	43951	98009	1046	13861	631	9296	71958	252,755
P11	2655	3054	17812	7674	2178	681	3668	631	930	67414	106,697
P12	4613	2387	0	698	137213	469	0	0	0	639	146,019
P13	1963	878	5937	27906	60984	446	6411	631	930	0	106,086
P14	2469	1413	0	4186	50094	639	9307	0	1859	14970	84,937
P15	6067	3313	0	0	261358	857	0	0	1859	8246	281,701
P16	4429	2863	47500	26510	39204	1754	21847	631	2789	14970	162,498
P17	0	281	0	0	0	0	12006	0	0	0	12,287
P18	908	1473	14844	9418	10890	442	2168	631	930	639	42,341
P19	2504	1493	0	26510	78407	1142	1504	0	930	14970	127,460
P20	3946	2236	2969	18487	71873	685	4154	0	1859	639	106,849
Média	3317	1991	7570	18732	67953	859	5539	284	2045	12167	120,458
DP	2715	1180	12613	16568	66677	500	5876	382	1922	20783	78,029
Mediana	2579	2023	1484	14650	56628	717	3716	-	1859	1277	106,773
IQR 25	1278	1279	-	4186	16879	463	1671	-	930	479	70,569
IQR 75	4631	2747	8164	30260	82763	1235	7135	631	2789	14970	150,138

UTI: unidade de terapia intensiva; IQR: intervalo interquartil; CCE: cateterismo cardíaco esquerdo; CCD: cateterismo cardíaco direito; DP: desvio padrão. *Outros procedimentos incluem hemodiálise, transfusão de hemocomponentes, biópsias endomiocárdicas, implantação de cardiodesfibrilador implantável e/ou terapia de ressincronização cardíaca, reparo mitral transcateter borda a borda, inserção de cateter venoso central de inserção periférica, angioplastia coronariana, cardioversão elétrica externa e implantação de balão intra-aórtico. Os custos são descritos em dólares internacionais (Int$).

O procedimento cardíaco mais frequente entre os pacientes estudados foi a cateterização do coração direito (CCD), realizada em 18 pacientes, com custo médio de Int$ 2.045 (DP 1.921). Outros procedimentos cardíacos invasivos e não invasivos incluíram hemodiálise, hemocomponentes, biópsias endomiocárdicas, implantação de cardiodesfibrilador implantável e/ou terapia de ressincronização cardíaca, reparo mitral transcateter borda a borda, inserção de cateter venoso central de inserção periférica, angioplastia coronariana, cardioversão elétrica externa e implantação de balão intra-aórtico. Nenhum dos pacientes do estudo recebeu dispositivo temporário de suporte circulatório mecânico além do balão intra-aórtico, uma vez que não eram elegíveis para transplante e a ponte para DAVE não era possível.

**Figura 3:**
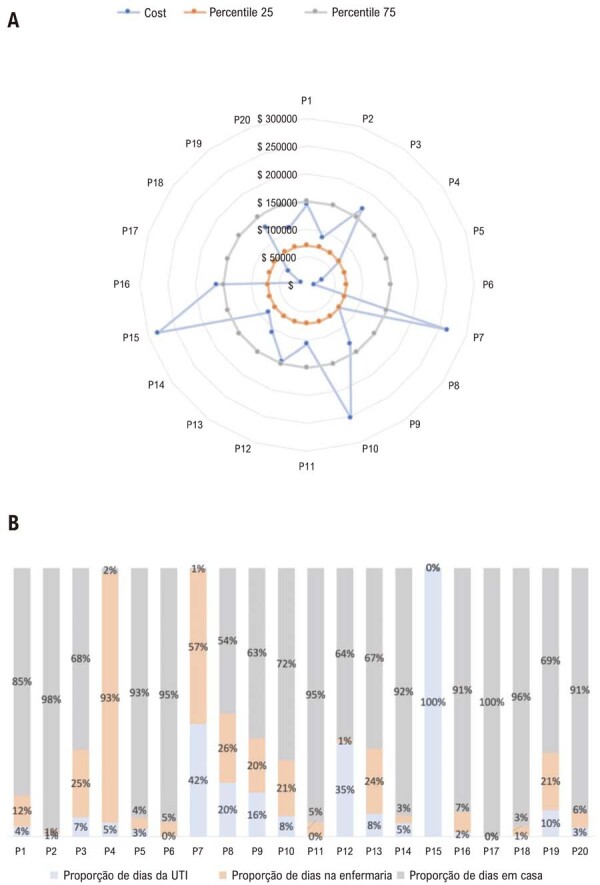
– A) Dispersão gráfica do custo total por paciente. B) Proporção de dias de internação por paciente durante o período do estudo. Percentual de dias que os pacientes permaneceram em leitos de Unidade de Terapia Intensiva (UTI) e não UTI durante o acompanhamento.

De forma geral, os pacientes com IC avançada apresentaram alta utilização de recursos de saúde e grande variabilidade nos custos, com mais pacientes registrando custos totais acima do percentil 75 da amostra total, e menos pacientes com valores abaixo do percentil 25. As Figuras 3A e 3B ilustram a variabilidade individual dos custos totais e o total de dias de internação em enfermaria ou UTI por paciente. Os pacientes permaneceram hospitalizados durante aproximadamente 30% do período do estudo (variação de 0 a 100%). O tamanho reduzido da amostra não permitiu comparações estatísticas entre pacientes com diferentes padrões de consumo de recursos.

**Figura 4:**
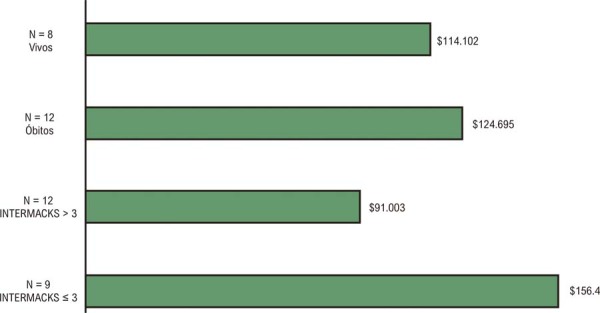
– Custo médio estimado por paciente de acordo com o status INTERMACS e desfecho.

Os custos totais médios por paciente foram analisados com base no perfil INTERMACS (INTERMACS ≤ 3 vs. > 3) no momento da indicação de DAVE, estendendo-se até o óbito ou a conclusão do estudo durante o período de acompanhamento ( Figura 4 ). De modo geral, os pacientes em pior condição clínica (INTERMACS ≤ 3) apresentaram custos aproximadamente 70% superiores aos daqueles em melhor estado clínico (p = 0,036). Por outro lado, os pacientes que faleceram durante o acompanhamento registraram custos totais semelhantes aos daqueles que permaneceram vivos até o final do estudo (p = 0,399). As maiores despesas com hospitalizações, procedimentos e exames foram os principais responsáveis pela variabilidade de custos entre os grupos de status INTERMACS. A mortalidade foi equivalente, com seis óbitos em cada um dos dois grupos INTERMACS. Pacientes com INTERMACS ≤ 3 permaneceram em média 112 dias (DP 53) hospitalizados, enquanto aqueles com INTERMACS > 3 permaneceram em média 62 dias (DP 52).

## Discussão

Este estudo relata os custos médicos diretos enfrentados por pacientes com IC avançada que não têm acesso a terapias avançadas (transplante cardíaco ou DAVE) no contexto do sistema público de saúde brasileiro. Entre os pacientes com doença grave, com alta mortalidade, o custo médio do tratamento foi elevado, alcançando Int$ 120 457 ao longo de 15 meses de acompanhamento. A Figura Central resume os principais achados.

As análises de custo assistencial em pacientes com IC avançada tratados clinicamente e que não são elegíveis para terapias de prolongamento da vida permanecem limitadas. Russo et al.,^19^ em 2008, avaliaram o braço de tratamento clínico do estudo *Randomized Evaluation of Mechanical Assistance for the Treatment of Congestive Heart Failure* e constataram que o custo médio do cuidado nos dois últimos anos de vida foi de USD$ 156 169, sendo 50,5% (USD$ 78.880,39) incorridos nos últimos seis meses.^19^ Nossos dados contemporâneos confirmam que o manejo clínico dessa população vulnerável continua caro, especialmente entre aqueles com maior gravidade clínica no momento em que a terapia com DAVE é considerada. Os custos assistenciais e a utilização de recursos de saúde aumentam entre os pacientes que evoluem para óbito, apesar do menor tempo de vida. Isso sugere que uma estratégia potencial para redução de custos pode envolver uma identificação mais precoce de pacientes com curso clínico pior. A falta de encaminhamento oportuno para terapias avançadas continua sendo um desafio, mas, conforme indica nossa análise de custos, pode representar uma oportunidade importante para intervenção.^10 , 20^

Os custos observados associados à IC avançada diferem substancialmente daqueles observados em pacientes com IC crônica e estável. Um estudo anterior que utilizou técnicas de microcustos para avaliar as despesas ambulatoriais constatou que o custo anual para pacientes na classe funcional I da *New York Heart Association* (NYHA) foi de Int$ 510, enquanto aqueles nas classes III/IV da NYHA apresentaram um custo de Int$ 1.334 por ano.^2 1^ Como o esperado, pacientes com doença mais grave demandaram mais recursos, com até 74% dos custos atribuídos a exames e procedimentos diagnósticos. Esses achados reforçam a necessidade de identificação precoce de pacientes de alto risco e sugerem que uma atenção maior à relação custo-efetividade das terapias avançadas pode ser necessária. Tais insights podem contribuir para políticas mais eficientes de alocação de recursos em sistemas de saúde públicos.

Além dos custos diretos relacionados ao tratamento da IC avançada, os custos indiretos também são relevantes, mas permanecem pouco quantificados na maioria dos cenários. A literatura sugere que os custos indiretos possam representar até 40% do custo total do manejo da doença.^22 , 23^ Como nosso estudo avaliou apenas os custos diretos com saúde, incluindo medicamentos, devemos ter cautela ao interpretar os dados apresentados. Ainda assim, é provável que nossas estimativas subestimem o real impacto social. Avaliações abrangentes do impacto econômico da IC sobre o sistema de saúde devem considerar fatores como perda de produtividade dos pacientes e/ou familiares, necessidade de cuidadores, aquisição de medicamentos e transporte para visitas frequentes a unidades de saúde para consultas e exames diagnósticos. Infelizmente, os custos indiretos são capturados de forma inadequada pelos sistemas eletrônicos de saúde e bases de dados administrativas atualmente disponíveis no Brasil. Portanto, nossos achados provavelmente subestimam o custo total associado ao manejo médico da IC avançada.

Nossas descobertas destacam a importância de realizar estudos de custo-efetividade para opções de tratamento destinadas à recuperação de pacientes com IC avançada. Estudos anteriores que avaliaram os dispositivos de assistência ventricular esquerda sugeriram uma relação custo-efetividade desfavorável; no entanto, essas análises estão agora desatualizadas, pois foram baseadas em sistemas considerados obsoletos.^24^ Com os avanços na tecnologia dos DAVEs e melhorias na gestão médica, observou-se uma redução significativa nas hospitalizações e na utilização geral de recursos de saúde.^25 , 26^

Uma análise recente realizada no Reino Unido utilizando o dispositivo HeartMate 3, em terapia de destino, demonstrou limiares de custo-efetividade significativos para os pagadores públicos. Este estudo relatou uma razão incremental de custo-efetividade de £47 361 por ano de vida ajustado pela qualidade (QALY) ganho, em comparação com a terapia médica otimizada, com razões ainda mais favoráveis observadas entre pacientes mais graves (INTERMACS < 3).^16^ Esperamos que estudos como o nosso possam contribuir com dados valiosos para futuras análises de custo-efetividade ou custo-utilidade baseadas em evidências do mundo real.

Uma limitação importante do nosso estudo está na sua concentração exclusiva nos custos diretos da assistência à saúde, com atenção limitada aos custos indiretos (conforme discutido anteriormente). Embora as hospitalizações tenham sido o principal fator financeiro para os pacientes incluídos nesta análise, outros custos diretos — como a administração de medicamentos em ambiente ambulatorial — não foram considerados. Além disso, os custos hospitalares diretos foram obtidos a partir de um único centro público localizado no sul do Brasil, e a estrutura financeira dessa instituição específica influenciou as estimativas de custo por unidade utilizadas em nossa análise.

Não conseguimos abordar as possíveis variações de custo entre diferentes sistemas hospitalares, a utilização individual de recursos e as alocações de custos entre os sistemas de pagamento público e privado. Este relatório representa uma referência do mundo real sobre os custos associados ao tratamento de pacientes com IC avançada que seriam elegíveis para terapia com DAVE. Esses pacientes frequentemente apresentam uma trajetória clínica heterogênea e dinâmica, com comorbidades que impactam significativamente o prognóstico e a evolução clínica.

Para padronizar o período do estudo, utilizamos o momento da indicação para terapias avançadas como ponto de referência. No entanto, muitos desses pacientes podem ter sido encaminhados tardiamente, uma vez que não foram considerados candidatos ao transplante cardíaco. É importante destacar que este é um estudo gerador de hipóteses, com uma amostra limitada, o que deve ser levado em consideração ao interpretar e aplicar os resultados. De modo geral, acreditamos que os custos reais do cuidado com a IC avançada podem ter sido subestimados.

## Conclusão

Este estudo apresenta uma avaliação abrangente dos custos diretos de saúde associados a pacientes com IC que são candidatos à terapia com DAVE como tratamento definitivo, mas que não conseguem receber essa intervenção que salva vidas. Nossos achados estabelecem as bases para futuras análises econômicas voltadas à avaliação de tecnologias de saúde existentes e emergentes para IC avançada, com o objetivo final de orientar modelos de cuidado eficazes e financeiramente sustentáveis.
