# Supplementary material for: Custos do Tratamento Clínico de Pacientes com Insuficiência Cardíaca Avançada na América Latina
Source: Arq Bras Cardiol. 2025 Nov 19;122(10):e20240813. [Article in Portuguese] doi: 10.36660/abc.20240813 (PMC12711218; doi:10.36660/abc.20240813)
Supplement: Translation

# Translation: The Cost of Medical Management in Advanced Heart Failure: A Latin American Perspective

**DOI:** 10.36660/abc.20240813i

**Authors:** Livia Adams Goldraich, Ana Paula Beck da Silva Etges, Laura Caroline Tavares Hastenteufel, Dayanna Machado Lemos, Andreas Zuckermann, Mandeep R. Mehra, Carisi Anne Polanczyk, Nadine Clausell

**Affiliations:** 1 Brazil Programa de Insuficiência Cardíaca Avançada e Transplante Cardíaco, Hospital de Clínicas de Porto Alegre, Porto Alegre, RS – Brazil; 2 Brazil Programa de Pós-Graduação em Cardiologia, Faculdade de Medicina, Universidade Federal do Rio Grande do Sul, Porto Alegre, RS – Brazil; 3 Brazil Instituto Nacional de Ciência e Tecnologia para Avaliação de Tecnologias em Saúde e Conselho Nacional de Desenvolvimento Científico e Tecnológico, Porto Alegre, RS – Brazil; 4 Brazil Programa de Pós-Graduação em Epidemiologia, Faculdade de Medicina, Universidade Federal do Rio Grande do Sul, Porto Alegre, RS – Brazil; 5 Austria Department of Cardiac Surgery, Medical University of Vienna, Vienna - Austria; 6 USA Brigham and Women’s Hospital and Harvard Medical School, Boston, MA – USA

**Keywords:** Costs and Cost Analysis, Heart Failure, Latin America

## Abstract

**Background:**

The financial burden faced by patients with advanced heart failure (HF) who are ineligible for heart transplantation or lack have access to advanced therapy with left ventricular assist devices (LVADs) has not been described in Latin American.

**Objective:**

To conduct a microcosting study in patients who were otherwise eligible for LVAD therapy who did not have access to it.

**Methods:**

We evaluated the direct costs of care in a cohort of advanced HF patients treated at a Brazilian public hospital who were candidates for LVAD therapy (and ineligible for transplantation) but lacked access to the procedure. A patient-level cost analysis was performed from the time LVAD need was identified until death or end of follow-up. The total cost per patient was calculated using the time-driven activity-based costing methodology and expressed in international dollars (Int$). Patients were stratified by disease severity and all-cause mortality to assess cost variability.

**Results:**

Twenty consecutive patients (mean age 51 years; 90% male) were followed for an average of 15 months, with an overall survival rate of 40%. The average cost per patient was Int$120,457 (SD Int$78,029). Hospitalizations represented the largest share of total costs, accounting for 72% (Figure 2). Patients with greater clinical severity (INTERMACS profile ≤ 3) incurred significantly higher costs (Int$156,457), nearly 70% more than those who did not require inotropic support (Int$91,003).

**Conclusions:**

This study highlights the financial burden associated with ongoing medical management of advanced HF in patients who would otherwise be eligible for LVAD therapy. These findings provide a basis for evaluating the cost-effectiveness of device-based, life-saving interventions in selected advanced HF populations.

## Introduction

Advanced heart failure (HF), a stage of disease marked by resistance to traditional pharmacological and non-pharmacological therapies, is associated with high morbidity and mortality, as well as increased healthcare resource utilization and costs for both individuals and society.^1^As populations age and benefit from improved therapies for early onset cardiovascular disease, the prevalence of advanced HF continues to rise, leading to a substantial societal burden and loss of productivity.^2^

While heart transplantation is now an established therapy for advanced HF, a substantial number of individuals are ineligible due to comorbidity or other reasons.^3^For these patients, a durable left ventricular assist device (LVAD) serves as a life-prolonging therapy; otherwise, they are often relegated to palliative care.^4^In the United States, 27,298 long-term LVADs were implanted between 2010 and 2019; in 2019 alone, the number of LVAD implants nearly matched the number of heart transplants performed nationwide, with the majority of cases classified as destination therapy for patients deemed ineligible for transplantation.^5 , 6^In Europe, approximately 500 individuals per year have received an LVAD over the last 10 years, and this number is steadily growing, due to the limited availability of donor organs.^7^

The outcomes for patients with long-term DAVE have improved substantially over time, largely due to better patient selection, enhanced clinical management, and the introduction of the fully magnetically levitated HeartMate 3 pump (Abbott, USA). Median survival now exceeds five years, particularly among patients who are otherwise ineligible for transplantation.^8 - 10^However, LVADs are not available in most low- and middle-income countries due to concerns of cost-effectiveness. In Brazil, the HeartMate 3 LVAD is accessible only through private insurance or philanthropic research initiatives.^11^The cost of care for patients with advanced HF who are unable to receive LVAD therapy remains largely unknown, despite its potential relevance for evaluating the cost-effectiveness of such interventions.

Healthcare resource utilization and the cost of care for patients with advanced HF who are treated without the possibility of salvage through LVAD or transplantation may represent a financial burden that is not readily recognized by individuals, healthcare systems, payors and governments. In selected cases, the judicious use of destination LVAD therapy may prove to be cost-effective.^11 , 12^Therefore, the present study, conducted within the Brazilian public healthcare system, aims to evaluate the longitudinal costs of medical management of advanced HF in patients who might have been eligible for destination LVAD therapy but lacked access to the procedure.

## Methods

We conducted a microcosting study (a financial estimation method that includes resource use and unit cost) to evaluate direct costs of a cohort of patients with advanced HF from the perspective of a public university hospital, in a regional reference center for advanced HF therapies. The sample consisted of patients with advanced HF who were ineligible to undergo a heart transplant but were eligible to receive an LVAD as an indication for destination therapy.^13^

### Population

We defined a cohort of consecutive adult patients with advanced-stage HF who met eligibility criteria for LVAD implantation as destination therapy but were unable to receive such treatment at our institution between January 2015, and May 2023. Advanced HF was defined through clinical evaluation, incorporating symptom burden, objective prognostic parameters and therapeutic interventions (such as inotropic therapy), as outlined by European and North-American cardiology societies and summarized by Truby and Rogers.^14^Exclusion criteria included those patients who underwent a heart transplant or a LVAD implantation, those with private insurance coverage, and those primarily followed at other institutions.

The clinical reasons for transplantation ineligibility were advanced age, severe pulmonary hypertension, obesity, active smoking, frailty and other comorbidities. Our program adheres to the guidelines of the International Society for Heart and Lung Transplantation regarding contraindications for transplantation.^15 , 16^Severe pulmonary hypertension was defined as pulmonary vascular resistance greater than 3 Wood units, not responsive to vasodilatory challenge. The flowchart of patients eligible for long-term LVAD therapy and selected for inclusion in the cost analysis is in Figure 1 .

**Figure 1:** – Flowchart of patients eligible for long-term left ventricular assistance device (LVAD) therapy at the reference center that were selected for cost analysis.

The data collection period for each patient began when the clinical team considered an indication for advanced therapy – either heart transplantation or LVAD – or at hospital admission for those already hospitalized on that date. The cost acquisition interval was censored at death, at the end of study follow-up on June 30, 2023 or at discharge for patients hospitalized on that date. Data sources included electronic discharge summaries, progress notes, outpatient clinic records, and heart transplant/DAVE assessment forms. Individual patient-level cost analysis included all hospital encounters, including clinic visits, diagnostic tests, procedures, and hospitalizations.

### Cost data

Cost components for THE overall cost analysis were obtained using the time-driven activity-based costing (TDABC) methodology.^17^In this approach, in which, resource consumption is measured based on the patient care cycle, considering both the unit cost of delivering care and the time required to perform each transaction or activity. Cost data related to personnel, infrastructure, procedures, and diagnostic tests (including imaging) were extracted from hospital administrative records, following the methodology used in previous studies focused on specific procedures and services provided to patients with advanced HF ( Table 1 ).

**Table 1:** – Sources of cost component information with updated values for the year of 2023

Healthcare provider	Salaries plus charges (Int$/h)	Source of information
Clinical cardiologist	73	Institutional financial department
Interventional cardiologist	86	Institutional financial department
Medical resident	6	Institutional financial department
Imaging cardiologist	82	Institutional financial department
Cardiovascular surgeon	82	Institutional financial department
Nurse	38	Institutional financial department
Nursing technician	21	Institutional financial department
Physiotherapist	36	Institutional financial department
Perfusionist	31	Institutional financial department
Anesthesiologist	93	Institutional financial department
**Infrastructure in hospitalization units**		
Unit	Fixed costs assigned to the unit (Int$)	Source of information
Catheterization laboratory	433 (hour)	Institutional financial department
Coronary care unit	2178 (day)	Institutional financial department
Intensive care unit	2969 (day)	Institutional financial department
Inpatient ward	349 (day)	Institutional financial department
Procedures and services*		
Procedure and/or service	Cost (Int$ per unit)	Source of information
PICC insertion	638	[28]
Central venous catheter insertion	259	[28]
NYHA II patient clinic consultation**	176	[29]
NYHA III and IV patient clinic consultation**	593	[29]
Day-hospital visit	76	[30]
Hemodialysis session	488	Institutional financial department
Coronary angioplasty	Materials 1664 + Professionals and room 1407	Institutional financial department
Left heart catheterization	Materials 240 + Professionals and room 390	Institutional financial department
Right heart catheterization	Materials 484 + Professionals and room 445	Institutional financial department
Intra-aortic balloon pump insertion	Materials 1281 + Professionals and room 678	Institutional financial department
Endomyocardial biopsy	Materials 743+ Professionals and room 390	Institutional financial department
ICD implantation	Materials + 11983 Professionals and room 1609	Institutional financial department
Electrical cardioversion	Materials 33 + Professionals and room 234	Institutional financial department
Mitral valve TEER	Materials 62015 + Professionals and room 2363	Institutional financial department

Int$: dólares internacionais; PICC: dos exames realizados no mesmo dia da visita. Int$: international dollars; PICC: for exams performed on the same day of the visit. NYHA: New York Heart Association; * Supplemental Table I describes the time spent by professionals and the cost of individual procedures. ** Outpatient visits include the cost of exams performed on the same day.

Medication costs were derived from patient-level consumption data obtained through the institutional administrative system. Procedural costs included materials, professional fees, and infrastructure. Interviews were performed to estimate the average time healthcare professionals dedicated to each procedure, consultation, outpatient clinic visit, and day-hospital care. Costs for individual hospital units – such as inpatient wards, intensive care units (ICUs), and catheterization laboratory – covered depreciation, energy, administrative supplies, professional salaries, and other expenses. For professional salaries, the exact staffing allocation for each unit was calculated, including the ICU area specifically designated for cardiology patients, which has a different staffing structure. The average monthly direct fixed cost for each unit was computed and divided by the unit’s monthly service capacity. For example, for the ICU, the capacity was determined based on the number of available beds: 45 beds operating 24 hours a day, 30 days, totaling 32,400 hours. The average daily cost was calculated by dividing the total cost attributed to the unit by the number of beds and working days. Hospitalization costs were then determined by multiplying the time each patient spent in a given unit by the corresponding cost per unit of time.

Cost components were calculated and aggregated to determine the total cost per patient. Descriptive cost analyses included mean, standard deviation (SD), median, and interquartile range. Patients were stratified by clinical variables – specifically, disease severity as classified by INTERMACS (Interagency Registry for Mechanically Assisted Circulatory Support) profiles – and by mortality outcome of death was performed to estimate cost variability. Also, an estimated annual cost *per* patient was calculated to represent the expected annual economic burden of patients with this clinical condition to the providers. As the study cohort included patients with varying follow-up durations, the total cost per patient was divided by the number of months of follow-up and then multiplied by 12. This adjustment is important to consider when applying the data to future economic models. All values were adjusted to the year 2023 using the Brazilian Broad National Consumer Price Index accumulated from the time the data was originally obtained (for costs extracted from scientific articles). These values are presented in international dollars (Int$) to reflect purchasing power parity, based on the World Bank conversion factor available at https://data.worldbank.org/indicator/PA.NUS.PPP (accessed on March 29, 2024). For reference, Int$ 1,000 is equivalent to US$ 1,000 and R$ 2,580. Comparisons between INTERMACS > 3 and INTERMACS ≤ 3 profiles, as well as between outcomes (alive vs. deceased) were performed using Student’s *t* test. All analyses were performed using Microsoft^®^Excel for Mac.

### Ethical Aspects

The research protocol was approved by the Institutional Research and Ethics Committee, and informed consent was waived due to the retrospective nature of the study. All researchers signed a confidentiality agreement to access individual data. The study was conducted independently and with full autonomy by the authors, without any interference from the sponsor.

## Results

The inclusion and exclusion criteria were met in 20 consecutive patients with advanced HF. Their mean age was 51 years, and 90% were male. Table 2 shows demographic and clinical characteristics of the study cohort. Three-quarter of patients were hospitalized at the time of inclusion and nearly half of them were in INTERMACS status ≤ 3 [these represent grades of stability on inotropic therapy from the least (1) to most (3) stable] when a destination LVAD was considered but could not be implanted. At the time of study inclusion, the proportion of guideline directed medical therapy for HF was relatively low (largely due to tolerability). SGLT2 inhibitors were not readily accessible in Brazil for HF treatment before 2022, hence use rates were low, however, utility in advanced HF remains uncertain as such patients were generally not included in the pivotal trials.^18^

**Table 2:** – Demographic and clinical characteristics of the study cohort

Demographics and comorbidities	N = 20
Sex, male	18 (90)
Age, years	51 ± 15
Diabetes	8 (40)
Atrial fibrillation or flutter	6 (30)
Creatinine, mg/dL	1.5 ± 0.6
**Characteristics of heart failure**	
Ischemic etiology	13 (65)
Left ventricular ejection fraction	0.21 [0.20- 0.24]
INTERMACS ≤ 3	9 (45)
Cardiac index, L/min/m^2 a^	2.2 ± 0.4
Pulmonary vascular resistance, Wood units^b^	3.6 ± 2.1
PASP, mmHg	56 ± 19
**Medications**	
ACE inhibitor/ ARB	4 (20)
Neprilysin inhibitor	7 (35)
Beta-blocker	15 (75)
Mineralocorticoid antagonist	14 (70)
SGLT2 inhibitor	5 (25)
Intravenous inotrope	9 (45)
Median follow-up time, months	15 [7.7 - 21.7]

Data expressed as number (percentage), mean ± standard deviation or median [interquartile range]. INTERMACS: Interagency Registry for Mechanically Assisted Circulatory Support; PASP: pulmonary artery systolic pressure; ACE: angiotensin-converting enzyme; ARB: angiotensin receptor blocker; SGLT2: sodium-glucose cotransporter-2.^a^Data available for 16 patients.^b^Data available for 17 patients.

The main contraindication to ineligibility for heart transplantation was pulmonary hypertension, followed by comorbidities. Overall survival was 40% at the end of a median of 15 months (min 4, max 36) of follow-up; eleven patients died due to progressive pump failure, and one from sudden death. Individual patients’ clinical severity characteristics and resource utilization information are described in Table 3 .

**Table 3:** – Clinical severity characteristics and resource utilization data for individual patients

Patient	Sex	Age, years	INTERMACS Status	Risk conditions for transplant	Year of LVAD indication	Follow-up time, months	Status at end of follow-up	Emergency visits and/or hospitalizations	Admission to ICU, days	Admission to ward and/or emergency room, days
**P1**	M	24	5	Pulmonary hypertension	2020	31	Death	0	36	109
P2	M	66	4	Obesity	2020	36	Alive	0	12	20
P3	M	69	5	Pulmonary hypertension	2022	15	Alive	2	32	123
P4	M	61	3	Pulmonary hypertension	2021	5	Death	0	8	139
P5	M	37	5	Pulmonary hypertension	2022	8	Alive	0	7	11
P6	M	66	5	Previous sternotomy, PAOD	2022	8	Alive	0	1	12
P7	M	43	3	Pulmonary hypertension	2023	7	Alive	0	88	120
P8	M	50	3	Pulmonary hypertension	2020	4	Death	0	24	31
P9	M	39	3	Pulmonary hypertension	2015	9	Death	0	44	56
P10	M	55	2	Pulmonary hypertension	2022	20	Alive	0	45	126
P11	M	64	4	COPD, PAOD	2020	14	Death	2	1	28
P12	M	51	2	Pulmonary hypertension	2018	6	Death	0	63	2
P13	F	49	3	Active smoking	2017	11	Death	1	28	82
P14	M	48	3	Pulmonary hypertension	2022	15	Alive	0	23	12
P15	M	14	3	Pulmonary hypertension	2020	4	Death	0	120	0
P16	F	63	5	Frailty, poorly controlled DM	2019	34	Death	0	18	92
P17	M	31	6	Cannabis-related pneumonitis	2021	11	Alive	0	0	0
P18	M	60	5	Obesity, poorly controlled DM	2017	27	Death	0	5	32
P19	M	58	4	Frailty, COPD	2019	12	Death	0	36	76
P20	M	62	4	Pulmonary hypertension	2016	32	Death	2	33	54

M: male; F: female; INTERMACS: Interagency Registry for Mechanically Assisted Circulatory Support; LVAD: left ventricular assist device; ICU: intensive care unit; PAOD: peripheral arterial occlusive disease; COPD: chronic obstructive lung disease; DM: diabetes mellitus. * P15 had a single, prolonged hospitalization since the initial clinical presentation.

### Cost analysis

The average cost per patient was Int$ 120,457 (SD 78,029), which represents a mean monthly cost of Int$ 8,030. Table 4 describes the cost composition *per* patient included in the study, and Figure 2 , the cost composition proportion. Patients spent 31 (SD 30) days in ICUs and 53 (SD 47) days in non-ICU units between the date of LVAD indication and the end of the study follow-up. Indeed, hospitalizations represented more than two-thirds of the total cost (56% in intensive care, and 16% in non-ICU care). Medications exclusively dispensed in-hospital accounted for 3% of the average overall cost.

**Table 4:** – Description of cost components, total cost, and annual cost per individual patient

Patient	Medications	Tests	ER visits	Admission to ward	Admission to ICU	Consultations	Day-hospital and clinics	LHC	RHC	Other cardiovascular and invasive procedures, hemodialysis and blood products	Overall cost
P1	9,146	2,709	5,937	37,324	78,407	1,350	5,507	0	2,789	1,277	144,446
P2	1,401	2,238	23,750	4,186	26,136	696	13,858	631	2,789	13,860	89,544
P3	5,128	2,653	26,719	39,765	69,696	1,545	6,259	0	1,859	15,509	169,133
P4	0	0	0	48,486	17,424	1,392	0	0	1,859	1,277	70,438
P5	326	849	2,969	3,488	15,246	308	1,727	0	2,789	0	27,701
P6	87	756	0	4,186	2,178	180	3,764	0	930	0	12,082
P7	4,413	1,473	0	41,858	191,663	1,622	2,964	1,263	2,789	16,338	264,381
P8	2,481	1,809	0	10,813	52,272	738	0	0	1,859	639	70,612
P9	4,683	3,055	2,969	19,185	95,831	1,196	1,778	631	1,859	0	131,188
P10	9,119	4,883	0	43,951	98,009	1,046	13,861	631	9,296	71,958	252,755
P11	2,655	3,054	17,812	7,674	2,178	681	3,668	631	930	67,414	106,697
P12	4,613	2,387	0	698	137,213	469	0	0	0	639	146,019
P13	1,963	878	5,937	27,906	60,984	446	6,411	631	930	0	106,086
P14	2,469	1,413	0	4,186	50,094	639	9,307	0	1,859	14,970	84,937
P15	6,067	3,313	0	0	261,358	857	0	0	1,859	8,246	281,701
P16	4,429	2,863	47,500	26,510	39,204	1,754	21,847	631	2,789	14,970	162,498
P17	0	281	0	0	0	0	12,006	0	0	0	12,287
P18	908	1,473	14,844	9,418	10,890	442	2,168	631	930	639	42,341
P19	2,504	1,493	0	26,510	78,407	1,142	1,504	0	930	14,970	127,460
P20	3,946	2,236	2,969	18,487	71,873	685	4,154	0	1,859	639	106,849
Mean	3,317	1,991	7,570	18,732	67,953	859	5,539	284	2,045	12,167	120,458
SD	2,715	1,180	12,613	16,568	66,677	500	5,876	382	1,922	20,783	78,029
Median	2,579	2,023	1,484	14,650	56,628	717	3,716	-	1,859	1,277	106,773
IQR 25	1,278	1,279	-	4,186	16,879	463	1,671	-	930	479	70,569
IQR 75	4,631	2,747	8,164	30,260	82,763	1,235	7,135	631	2,789	14,970	150,138

ER: emergency room; ICU: intensive care unit; IQR: interquartile range; LHC: left heart catheterization; RHC: right heart catheterization; SD: standard deviation. *Other procedures include hemodialysis, blood products, endomyocardial biopsies, implantable cardioverter defibrillator and/or cardiac resynchronization therapy insertion, transcatheter edge-to-edge mitral valve repair, peripherally inserted central catheter insertion, coronary angioplasty, external electrical cardioversion and intra-aortic balloon pump implantation. Costs are described in international dollars (Int$).

The most frequent cardiac procedure among studied patients was right heart catheterization (RHC) (18 patients), with an average cost of Int$ 2,045 (SD 1,921). Other invasive and non-invasive cardiac procedures included hemodialysis, blood products, endomyocardial biopsies, implantable cardioverter defibrillator and/or cardiac resynchronization therapy insertion, transcatheter edge-to-edge mitral valve repair, peripherally inserted central catheter insertion, coronary angioplasty, external electrical cardioversion and intra-aortic balloon pump (IABP) implantation. None of the study patients received a temporary mechanical circulatory support device other than IABP since they were not transplant-eligible, and bridging to a LVAD was not possible.

Overall, patients with advanced HF demonstrated high health care resource utilization and cost variability, with more patients registering a total cost higher than the percentile 75 of the total sample, and less patients with cost values lower than the percentile 25. Figures 3A and 3B illustrate the individual variability of total costs and the individual total of days hospitalized in the ward or in an ICU. Patients spent around 30% of the study period hospitalized (range 0-100%). The small sample size did not allow statistical comparisons among patients with distinct resource consumption patterns.

Average total costs per patient were further analyzed based on the INTERMACS profile (INTERMACS ≤ 3 vs. > 3) at the time of LVAD indication, continuing through to either death or study completion during the follow-up period ( Figure 4 ). Overall, patients in poorer clinical condition (INTERMACS ≤ 3) incurred costs nearly 70% higher than those in better status (p = 0.036). Meanwhile, patients who died during follow-up had similar total costs compared to those who survived until the end of the study (p= 0.399). Higher expenses with hospitalizations, procedures, and tests largely drove the cost variability between groups of INTERMACS status. The mortality was equivalent, as six patients died in each of the two INTERMACS groups. Patients with INTERMACS ≤ 3 spent an average of 112 (SD 53) days in the hospital, while those with INTERMACS > 3 spent an average of 62 (SD 52) days.

**Figure 4:** – Estimated mean cost per patient according to INTERMACS status and outcome.

## Discussion

This study reports the direct medical costs encountered among patients with advanced HF who are unable to receive advanced therapies (heart transplantation or LVAD) within the Brazilian public health system context. Among severely ill patients with high mortality the average treatment cost was high, reaching Int$ 120,457 in a 15-month follow-up period. The Central Illustration summarizes the main findings.

Cost of care analyses in medically managed advanced HF patients who are not eligible for life-prolonging therapy remain limited. Russo et al.,^19^in 2008, evaluated the medical management arm of the Randomized Evaluation of Mechanical Assistance for the Treatment of Congestive HF trial, and found that the average cost of care during the final two years of life was USD$156,169, with 50.5% (USD$78,880.39) incurred in the last six months.^19^Our contemporary data confirms that medical management for this vulnerable patient population continues to be costly, particularly among those with greater clinical severity at the time LVAD therapy is considered. Healthcare costs and resource utilization escalate in patients who die, despite their shorter lifespan. This suggests that one potential strategy to reduce costs may involve earlier identification of patients with a markedly declining clinical trajectory. The lack of timely referral for advanced therapies remains a challenge, but as our cost analysis indicates, it may represent a critical opportunity for intervention.^10 , 20^

The observed costs associated with advanced HF differ substantially from those incurred by patients with chronic, stable HF. A previous study employing microcosting techniques to assess ambulatory expenses found that the annual cost for patients in New York Heart Association (NYHA) functional class I was Int$ 510, whereas those in NYHA class III/IV accounted for Int$ 1334 per year.^21^As expected, patients with more severe disease were more resource-intensive, with up to 74% of their costs attributed to diagnostic tests and procedures. These findings further support the need for earlier identification of high-risk patients and suggest that greater attention to the cost-effectiveness of advanced therapies may be warranted. Such insights could inform more efficient healthcare resource allocation policies, particularly within publicly funded systems.

In addition to the direct costs related to the treatment of advanced HF, indirect costs are also relevant but remain poorly quantified in most scenarios. The literature suggests that indirect costs may account for up to 40% of the total cost of disease management.^22 , 23^Since our study assessed only direct healthcare costs, including medications, we should be cautious when interpreting the present data. Nonetheless, it is likely that our estimates underestimate the actual societal burden. Comprehensive evaluations of the economic impact of HF on the healthcare system should consider factors such as loss of productivity for patients and/or family members, the need for caregivers, medication acquisition, and transportation for frequent visits to healthcare facilities for consultations and diagnostic tests. Unfortunately, indirect costs are inadequately captured by current Brazilian electronic health systems and administrative databases. Therefore, our findings likely underrepresent the total cost associated with the medical management of advanced HF.

Our findings highlight the importance of conducting cost-effectiveness studies for treatment options aimed at salvaging patients with advanced HF. Previous studies evaluating LVADs suggested unfavorable cost-effectiveness; however, these analyses are now outdated, as they were based on legacy systems.^24^With advancements in LVAD technology and improvements in medical management, there has been a notable reduction in hospitalizations and overall healthcare resource utilization.^25 , 26^

A recent analysis conducted in the United Kingdom using the contemporary HeartMate 3 LVAD for destination therapy demonstrated cost-effectiveness thresholds that are meaningful for public payers. This study reported an incremental cost-effectiveness ratio (ICER) of £47,361 per quality-adjusted life year (QALY) gained compared to optimal medical therapy, with even more favorable ratios observed among sicker patients (INTERMACS < 3).^16^We hope that studies like ours can contribute valuable data to future cost-effectiveness or cost-utility analyses based on real-world evidence.

A major limitation of our study lies in its exclusive focus on direct healthcare costs, with limited attention to indirect costs (as previously discussed). Although hospitalizations were the primary financial driver for the patients included in this analysis, other direct costs—such as outpatient medication administration—were not accounted for. Additionally, the direct hospital costs were derived from a single publicly funded center in southern Brazil, and the financial structure of this specific institution influenced the unit cost estimates used in our analysis.

We were unable to address potential cost variations across different hospital systems, individual resource utilization, and cost allocations between private and public payer systems. This report provides a real-world reference for the costs associated with treating patients with advanced HF who are otherwise eligible for LVAD therapy. These patients often present with a heterogeneous and dynamic disease trajectory, with comorbidities significantly impacting prognosis and clinical outcomes.

To standardize the study period, we used the time of indication for advanced therapies as a reference point. However, many patients may have been referred late in their disease course, as they were not considered candidates for heart transplantation. Importantly, this is a hypothesis-generating study with a limited sample size, which should be considered when interpreting and applying the findings. Overall, we believe that the actual costs of care for advanced HF may have been underestimated.

## Conclusion

This study presents a comprehensive evaluation of direct healthcare costs associated with patients with advanced HF who are candidates for destination LVAD therapy but are unable to receive this life-saving treatment. Our findings lay the groundwork for future economic analyses aimed at evaluating both existing and emerging healthcare technologies for advanced HF, with the ultimate goal of informing financially sustainable models of effective care.

**Figure 2:** – Proportions of cost components for the overall patient cohort. ICU: intensive care unit; Int$, international dollars. Other procedures include hemodialysis, blood products, endomyocardial biopsies, implantable cardioverter defibrillator and/or cardiac resynchronization therapy insertion, transcatheter edge-to-edge mitral valve repair, peripherally inserted central catheter insertion, coronary angioplasty, external electrical cardioversion and intra-aortic balloon pump implantation.

**Figure 3:** – A) Graphical dispersion of total cost per patient. B) Proportion of hospitalization days per patient during the study period. Percentage of days patients spent in intensive care unit (ICU) and non-ICU beds during their follow-up.
